# A switching method for traveling/standing wave transportation modes in two-dimensional acoustic fields using a dual-transducer support structure

**DOI:** 10.1016/j.ultsonch.2023.106724

**Published:** 2023-12-09

**Authors:** Guanyu Mu, Huijuan Dong, Tong Sun, Kenneth T.V. Grattan, Zhiguang Wu, Jie Zhao

**Affiliations:** a State Key Laboratory of Robotics and System, Harbin Institute of Technology, Harbin 150001, Heilongjiang Province, China; b School of Science & Technology, City, University of London, London EC1V 0HB, United Kingdom

**Keywords:** Non-contact transportation, Dual-transducer support structure, Two-dimensional traveling wave, Two-dimensional standing wave, Temporal phase shift

## Abstract

The dual-transducer support structure discussed has the advantages of a simple structure and low cost, as well as allowing for the use of both Traveling-Wave (TW) and Standing-Wave (SW) acoustic transportation, supporting its use in pharmaceutical and biochemical analysis, for example. By adjusting the distance between the vibrating plate and the reflector which forms SW field in the *y* direction, the control of the position of the SW nodes or the TW component along the *x* direction allows the formation of a Two-Dimensional Standing Wave (2D-SW) or a Traveling Wave (TW) acoustic field, and these could be used for transportation in the *x* direction. It has been found that the *x* position of the SW nodes can be adjusted through changing the temporal phase shift, θ, which permits multiple objects to be transported using the 2D-SW mode. By comparison, TWs in the opposite direction could be generated at a pair of specific temporal phase shifts, allowing fast transportation using the TW mode. In this research work, an experiment has been carried out to transport polystyrene spheres using the two modes by programming the temporal phase shift, θ, this illustrating that precise position control of the multiple objects transported was possible using the 2D-SW mode, while high-speed transportation (up to 540 mm/s) was realized using the TW mode, showing that the dual-transducer support structure could be used effectively for accurate and fast transportation. As a fully non-contact method, the dual-transducer support structure can be seen to work in the 2D-SW mode for reaction synthesis or detection applications, and also in TW mode for rapid sample transportation applications.

## 1 Introduction

Acoustic levitation and transportation have many potential applications in metallic solidification [1], contactless transport of matter [2], [3], drop dynamics [4] and analytical chemistry [5], [6], [7] because a wide range of materials are supported compared to other levitation techniques. The use of acoustic transportation can not only levitate objects non-contactly to avoid contamination, but also facilitates enhancement the solubility of drugs [8], accelerating chemical reaction and crystallization process. To date, two supporting structures for the transducers are mainly used in acoustic transportation – one being the array structure composed of multiple transducers [2], [9], [10], [11] and the other the simple dual-transducer support structure [12], [13]. The array structure only permits standing wave (SW) transportation of materials or samples [2], [9], [10], [11], achieved through moving the position of the SW nodes, by adjusting the amplitude [2], [10] or phase [9], [14], [15] using the adjacent transducers. Although it is easy to obtain high positional accuracy using the array structure which facilitates chemical reaction or detection, the distance covered and speed of the transportation achieved are limited and a large number of transducers is required to structure an array, which increases the complexity of the power circuit used and thus the total cost.

By comparison, when using a dual-transducer support structure [12], [13], only two transducers are used to form either the standing wave (SW) [13], [16], [17] or the traveling wave (TW) [18], [19], [20] and a mixture of SW/TW. The major advantage of this structure is that it can not only use the SW mode, which offer high positional accuracy due to their radial stability effect, but also allow TW mode, to fulfill the need for rapid transportation. Depending on the practical requirements, theoretically, an unlimited transportation distance could be realized by aligning multiple sets of the dual-transducer structure.

There are two methods for generating TWs, i.e., the “excitation-absorption” and “excitation-excitation” methods are used. To use the dual-transducer support structure, the excitation-absorption [21], [22], [23] method can be used to generate TWs, which can allow fast operation of the levitated object, where the speed can reach up to 2.3 m/s [22]. However, an absorbing material is required at one end of the structure to reduce vibrations, which results in the difficulty of acoustic impedance matching, the energy absorbed from the vibration will also cause thermal energy losses [22]. In addition, the excitation-absorption method lacks the capacity to programmatically control the traveling/standing waves ratio, which is crucial for adjusting the speed of transportation. As a result, the “excitation-absorption” mode lacks the capability to gradually decelerate high-speed workpieces or pharmaceuticals at the end of the traveling wave transportation process. This limitation can result in damage or contamination, thereby limiting the industrial applications of acoustic levitation transport devices.

Alternatively, the “excitation-excitation” method shows no such problems, as it can be programmed to adjust the ratio between the TW and the SW used, as well as the direction of the TW [24], meaning that it could work on both the SW and TW modes. Although the “excitation-excitation” method has already been successfully applied in near-field acoustic levitation [25], [26], its application to SW field levitation (using TW transportation) has not yet been reported. Implementing the “excitation-excitation” method in a SW acoustic field will facilitate both fast transportation (using TWs) and precise transportation (using SWs). This capability allows gradually decelerate and direction control of the transported objects through the dual-transducer support structure. Consequently, developing a switching method for TW/SW transportation modes using this structure becomes a technical challenge, which currently limits its industrial application.

In this study, both the TW and SW modes were employed in the dual-transducer support structure. To do so, the conditions for forming TWs/SWs on the vibrating plate of the dual-transducer support structure were first theoretically deduced, and then the corresponding TW/SW acoustic field was measured. Following that, a series of experiments was carried out to compare the motion characteristics of the two transportation modes. Not only could more accurately positioning of the multiple objects be realized simultaneously using SWs on the dual-transducer support structure, in the same way as the array structure; but also fast, long-distance transportation could be achieved using TWs. This study aims to achieve theoretical control of the TW/SW modes on the dual-transducer support structure. This control provides a direct and programmable method for managing transportation in future industrial applications of acoustic transport.

## 2 Transportation theory using dual-transducer support structure

### 2.1 Principle of levitation in two-dimensional acoustic field

**Fig. 1:**
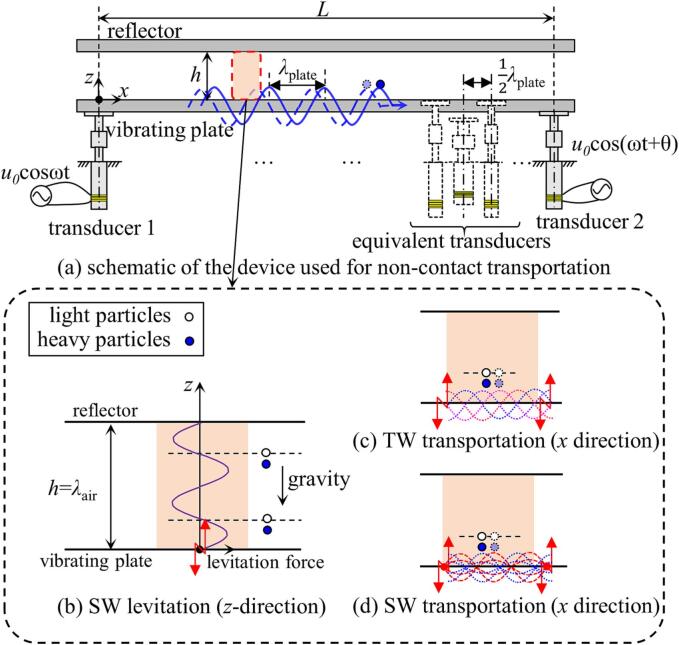
Schematic of the distribution of a two-dimensional SW acoustic field in a dual-transducer support structure.

The schematic diagram of the dual-transducer support structure is shown in Fig. 1(a), where a Cartesian coordinate (*x* and *z* axes) was established. Ths structure is consisted of two transducers placed at a distance, *L*, a vibrating plate connecting the two transducers by bolts, and a separate reflective surface. When the distance between the vibrating plate and the reflecting surface, *h*, is an integer multiple of the half-wavelength of the sound wave in air, *λ_z_/*2, a SW acoustic field will be generated along the *z* direction, as shown in Fig. 1(b). The distribution of the acoustic radiation force acting on the levitated sphere is illustrated in Fig. 1(b), as can be calculated using Gor’kov’s [27] or King’s [28] theory.

When an excitation signal, at the resonant frequency of the transducers, ω, and having the same amplitude *u_0_*, but with a phase shift, *θ*, is applied to the transducers, a vibration will occur along the vibrating plate in the *x* direction, as shown by the blue curve in Fig. 1(a). It can be seen from the figure that there are multiple peaks in the vibration distribution that can be used to levitate objects, and the distance between each peak is the half wavelength, λ_plate_/2 = *c*/2*f*, (where *c* is the speed of sound on the vibrating plate and *f* = 2πω is the excitation frequency). These vibration peaks seen are similar to the vibration distribution formed by an array of equivalent transducers arranged in the *x* direction [10], [16]. So, when the SW acoustic field in the *z* direction is generated by each equivalent transducer at the same time, the two-dimensional acoustic field can be formed in the *x-z* plane to transport the objects. Therefore, by replacing the transducer array, by using the simple dual-transducer support structure, the complexity of the mechanical structure and of the power control unit can be reduced.

Using such a structure, transportation of objects based on the Two-Dimensional Standing Wave (2D-SW) mode becomes possible. In the 2D-SW mode, adjustment of the temporal phase difference of the excitation transducers is required at all the time to move these equivalent transducers horizontally (in the *x* direction), while the position of the SW nodes in the acoustic field changes as well, this resulting in a positional shift of the levitated object. Additionally, the Traveling Wave (TW) mode can also be adopted into this structure, and this is done by applying a specific fixed temporal phase difference to the two transducers, so that the superimposed vibrations would form the TW vibration along the *x* direction. Compared to the SW transportation mode, the TW mode is able to move the objects concerned more quickly. In summary, the dual-transducer support structure offers the flexibility to control the ratio of SW and TW by adjusting the value of θ. The SW mode relies on continuous temporal phase adjustment, while the TW mode employs a fixed phase difference for faster transportation. This approach simplifies the mechanical structure complexity and reduces the requirements for the power control unit compared to an array structure.

In this study, a switching method between the 2D-SW and the TW transportation modes has been described, based on a dual-transducer support structure. Schematics of the two modes used are shown in Fig. 1(c) and (d) respectively, where it can be seen that in a 2D acoustic field, the object was transported along the *x* direction, using the SW or TW. However, in the *z* direction, Standing Wave Acoustic Levitation (SWAL) was always used. The figure shows an example of two layers of levitation positions, and the actual levitation position of the object (red) seen was slightly under the sound pressure node (white) because of the effect of gravity.

### 2.2 Principle of TW transportation based on a fixed specific temporal phase difference

The TW transportation mode offers several advantages, including high levitation height, as well as stable and rapid transportation. The “excitation-excitation” method adopted in this work is capable of programmable control of the direction of the TW on the dual-transducer support structure. It is worth noting that, in these reports [25], [26], TW transportation using the “excitation-excitation” method was only applied to near-field acoustic levitation.

To understand the flexural TW vibration along the *x*-direction of the vibrating plate under a specific temporal phase shift, two electrical signals, having the same amplitude and frequency, U_0_ and ω respectively, but with a phase shift, θ, are applied to the two transducers (which are shown in Fig. 1 (a). The equation for the displacement due to vibration of the plate in the *z* direction, *w*(x,t) (i.e., at time, t, and position, x) can be written as [24]:

(1) w(x,t)=cosωtcoskx+cos(ωt+θ)cos(kx+φ)

where *ω* is the angular frequency, *φ* is the spatial phase difference between the two transducers, given by *φ* = *k*(*L*-*nλ_plate_*), *n* is an integer, and λ*_plate_* and *k* are the vibrational wavelength and the wave number respectively. Therefore, the vibrational displacement is related to the material used (the Young's modulus, E, the density, ρ, and the Poisson ratio, μ), the shape of the plate (the thickness and the length), and the excitation signals (the frequency, *f*). When the TW is formed, the maximum and minimum amplitudes of the vibrational displacement values, *A*_max_(*θ*) and *A*_min_(*θ*), on the vibrating plate should be equal, so the two extreme positions were required and they can be derived from Eq. (1) as shown:

(2) $$\left\{\begin{array}{c} A_{\max}\left(\theta \right)=max\left(\left|2\sin\frac{\varphi }{2}\sin\frac{\theta }{2}\right|,\left|2\cos\frac{\varphi }{2}\cos\frac{\theta }{2}\right|\right)\\ A_{\min}\left(\theta \right)=\min\left(\left|2\sin\frac{\varphi }{2}\sin\frac{\theta }{2}\right|,\left|2\cos\frac{\varphi }{2}\cos\frac{\theta }{2}\right|\right) \end{array}\right)$$

Therefore, by applying the condition for the TW, *A*_max_(*θ*) = *A*_min_(*θ*), the two temporal phase shifts, *θ*_1_ and *θ*_2_, can be calculated as follows (from our previous work) [24], this being the condition of generating pure TWs:

(3) $$\left\{\begin{array}{c} \theta_{1}=\pi \;\text{-}\;\varphi ,\theta_{2}=\pi +\varphi \left(0<\varphi <\pi \right)\\ \theta_{1}=\varphi \;\text{-}\;\pi ,\theta_{2}\;\text{= 3}\pi \;\text{-}\;\varphi \left(\pi <\varphi <2\pi \right) \end{array}\right)$$

From Eq. (3), it is clear that the range of the values of φ will affect the values of *θ*_1_ and *θ*_2_. When the spatial phase difference satisfies the condition 0 < φ < π, adjusting the temporal phase shifts to *θ*_1_ = π - *φ* or *θ*_2_ = π + *φ* will allow the propagation of the TW. Similarly, when the value of φ lies within the range π < φ < 2π, the TW temporal phase shifts become *θ*_1_ = φ - π or *θ*_2_ = 3π - φ. Consequently, the relationship between the programmable value, *θ*, and the static structural value, *φ*, given in Eq. (3), allows for the generation of TWs regardless of the supporting distance, *L*, of the vibrating plate.

Therefore, if the temporal phase shifts were programmed to values *θ_1_* or *θ_2_*, which depend on the value of *φ*, the TW acoustic field would be generated along the *x* axis, but in the opposite direction. The normalized vibrational displacement of the two TWs, $\hat{w}$(*x,t*), can be expressed as [24]:

(4) $$\hat{w}\left(x,t\right)=\left\{\begin{array}{c} \text{sin}\varphi sin(\pm \omega t-kx+\varphi )(0<\varphi <\pi )\\ \text{sin}\varphi sin(\mp \omega t-kx+\varphi )(\pi <\varphi <2\pi ) \end{array}\right)$$

According to Eqs. (3), (4), once the TW is formed at *θ_1_* or *θ_2_*, then both the propagation direction and the amplitude of the TW can be determined by the value of *φ*.

As a result, in the TW transportation mode, the TW acoustic field along the *x* direction can be used for transportation of objects. Simultaneously, a SW acoustic field in the *z* direction can be used to levitate objects, by setting the height between the vibrating plate and the reflector to be the value of an integer multiple of 0.5λ_air_ (*h* = nλ_air_/2), as shown in Fig. 1(c). The TW mode can be seen as an equivalent transducer array moving at the speed of the TW, facilitating the transportation of the object.

### 2.3 Principle of 2D-SW transportation based on variable temporal phase shift

The TW transportation mode has the advantage of allowing fast and continuous transportation while, by comparison, the 2D-SW transportation mode can provide greater stability and more precise positioning, due to the presence of the acoustic potential well, as its position is only associated with θ. Therefore, establishing the relationship between θ and the position of the SW node in the *x* direction is required to realize the 2D-SW transportation on the dual-supported transducer structure. To do so, the vibration displacement of the plate, *w(x,t)*, as described in Eq. (1), can be decomposed into a TW component and a SW component as shown below:

(5) $$w\left(x,t\right)=\underset{\text{SW component}}{\underbrace{\cos\omega t\cos kx+\cos\omega t\cos(kx+\theta +\varphi )}}+\underset{\text{TW component}}{\underbrace{\sin(\theta )\sin(kx-\omega t+\varphi )}}$$

The SW component is mainy focused in the 2D-SW transport mode and it can be further simplified from Eq. (5):

(6) $$w_{\mathrm{SW}}\left(x,t\right)=2\cos\frac{\theta +\varphi }{2}\cos\omega t\cos\left(kx+\frac{\theta +\varphi }{2}\right)$$

It can be seen from Eq. (6) that the position of the SW node is $x_{s}=\left(\frac{n}{2}+\frac{\lambda }{4}-\frac{\theta +\varphi }{4\pi }\right)\lambda$, which changes linearly with the value of *θ*. The SW node (equivalent transducer) moves λ_plate_/2 to the negative direction of *x*, when *θ* changes from 0 to 2π, where it can be noted that the two TW temporal phases, *θ_1_* and *θ_2_*, should be bypassed to avoid the TWs. Similarly, if *θ* changes from 0 to −2π, the SW node moves to the *x +* direction and in this way, 2D-SW transportation of the object can be realized.

Based on the transportation principles of TWs and 2D-SWs, it is clear that when the temporal phase shift, θ, is adjusted, not only does the position of the SW node change, but also the ratio between the SW and the TW changes. Thus when the value of θ increases from 0° to 360°, the SW node position moves by λ_plate_/2 compared with the initial state. During this process, except for the cases where θ are 0° and 180°, (where only the SW is formed on the vibrating plate), the TW component is always present. Further, the TW component reaches its maximum at values of both *θ_1_* and *θ_2_*, determined from Eqs. (2), (3), which need to be avoided using the 2D-SW transportation mode because transportation using the TW is not desired. However, the use of the TW transportation mode is based on the two fixed specific temporal phase shifts, *θ_1_* and *θ_2_*, where then two TWs in the opposite direction are used to transport objects using this mode.

By varying θ to become 360°, the position of the SW node changes by half a wavelength. As a result, by adjusting θ appropriately, the two transportation modes, TW and 2D-SW can be both realized on the dual-transducer support structure, a situation which can not only transport the object more quickly, but also allow realizing accurate positional control.

## 3 FEM simulation of the TW and 2D-SW acoustic field

### 3.1 Generation of TW at a pair of specific temporal phase shift

**Fig. 2:**
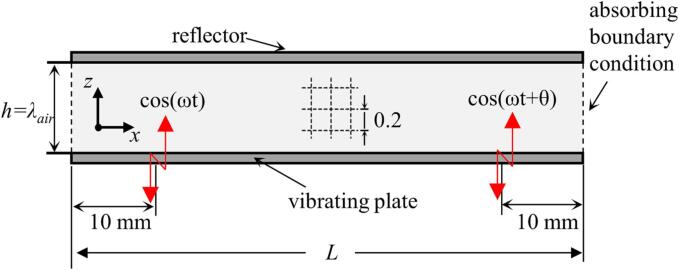
Schematic of the finite element model used for simulation of the acoustic field.

In order to verify the TW mode, based on a specific temporal phase shift, a finite element (FE) model of the acoustic field of the dual-transducer support structure was developed. To balance accuracy and simplicity in the simulation, a 2D model with a mesh size of 0.2 mm was used, as shown in Fig. 2. The vibrating plate in this model had a length (*L*) of 290 mm, a width of 60 mm, and a thickness of 3 mm. Two displacements sources are positioned at 10 mm from each end of the vibrating plate and are used to replace the excitation of the two transducers. These sources have identical amplitude and frequency, but there is a phase shift between them. The medium between the vibrating plate is air and the reflector used has the same length as the vibrating plate, with its thickness equivalent to one wavelength, λ_air_. The upper boundary of the medium (air) was set as a wall condition to maximize the reflection of the sound wave using the reflector. At the left and right boundaries of the air medium, a plane wave radiation condition was applied to allow minimal reflection of the outgoing sound propagation.

**Fig. 3:**
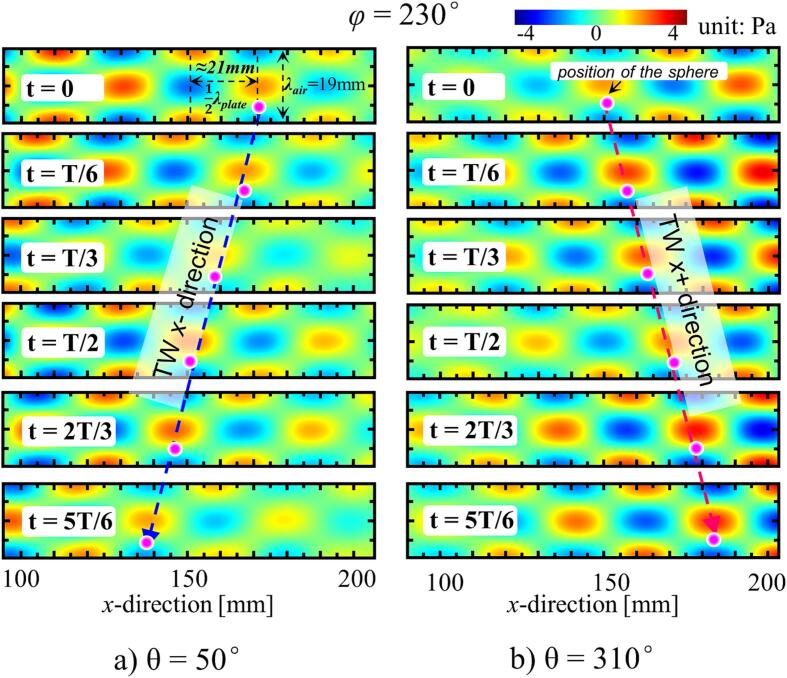
FEA results of the TW acoustic field in the *x +* and *x-* direction under specific temporal phase shift *θ*_1_ = 50° and *θ*_2_ = 310°, respectively (*f* = 18.0 kHz).

Prior to the simulation of the TW, it is essential to select an excitation frequency that falls between the two adjacent resonant frequencies. To do so, a modal analysis was performed, and the resonant frequencies of the plate were determined to be 17.45 kHz (corresponding to 7.5 wavelengths) and 19.87 kHz (corresponding to 8 wavelengths). Consequently, an excitation frequency of 18 kHz was chosen, corresponding to a spatial phase shift between 180° and 360° (180°<φ < 360°). In the simulation carried out, the distance between the vibrating plate and the reflector was set to *λ*_air_ = 19 mm and the value of θ was varied from 0° to 360°, with intervals of 5° used. The results obtained indicated that the TWs were formed in the *x*-direction when *θ*_1_ was set to 50° and *θ*_2_ was set to 310°. The results of the corresponding sound pressure distribution obtained at the center of the dual-transducer support structure (100 mm < *x* < 200 mm) are shown in Fig. 3. The results of the simulation carried out clearly show the formation of a SW in the *z*-direction, where the TW propagates in the *x* + direction (*θ*_1_ = 50°) and *x*- direction (*θ*_2_ = 310°), indicating that the spatial phase difference falls within the range of 180° < φ < 360°. Therefore, the characteristics of the TW mode based on the specific temporal phase shifts, *θ*_1_ and *θ*_2_, have been successfully verified. Additionally, the corresponding spatial phase difference can be determined to be φ = 230°, this having been calculated using Eq. (4) under an excitation frequency of 18 kHz.

Although the use of the TW mode enables the direction to be easily adjusted to allow rapid transportation on the dual-transducer support structure, achieving precise positioning in this mode can be challenging. In contrast, using the 2D-SW mode, inaccuracies in positioning could be effectively avoided. By adjusting the step size of *θ*, it is possible to switch between the TW and 2D-SW modes on the dual-transducer support structure. This allows fast transportation using the TW mode and accurate positioning using the 2D-SW mode, while offering a flexible approach for the differing requirements of a range of applications.

### 3.2 Moving position of the 2D-SW nodes by adjusting the temporal phase shift

To verify the 2D-SW mode through the adjustment of temporal phase shift, a 600 mm long and 3 mm thick duralumin plate was used in the model to simulate the acoustic radiation force of the acoustic field, based on the results of the FEA model used. During the transportation process, it is necessary to avoid the TW condition occurring, which means that the value of *θ* should not be equal to *θ*_1_ or *θ*_2_. To do so, the temporal phase was changed from 0° to 360°, intervals of 60° used.

**Fig. 4:**
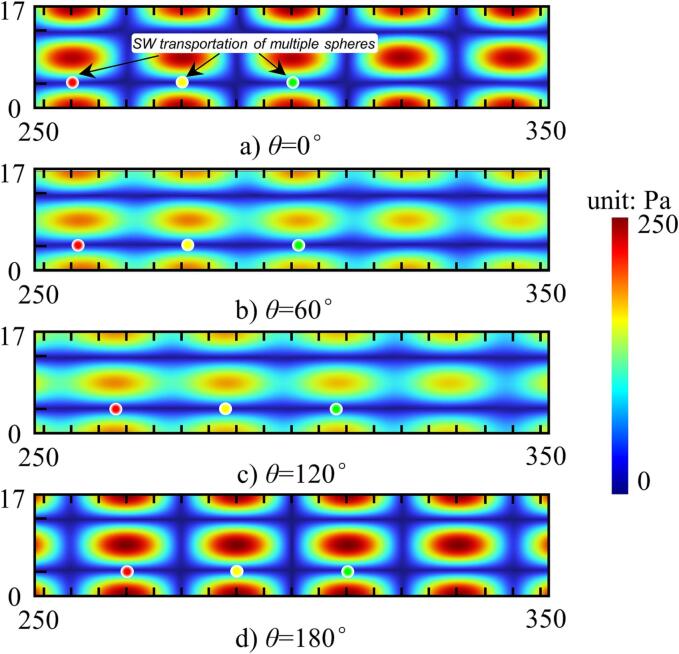
FEA results of the SW acoustic field, where the nodes move to the *x* + direction (*f* = 18.0 kHz, and *θ* was changed from 0° to 180°, with an interval of 60° used).

The results of the FE analysis for the sound pressure distribution are presented in Fig. 4, where the colored dots represent the positions of multiple sound pressure nodes. It can be observed that these positions moved progressively from left to right, as the temporal phase was changed. Moreover, throughout the range of temporal phases, potential wells were seen to exist, providing a stable levitation force and this enables the transportation of multiple objects simultaneously. In this way, the transportation of levitated objects using the 2D-SW mode by changing the node position by adjusting the temporal phase shift is possible.

## 4 System configuration

**Fig. 5:**
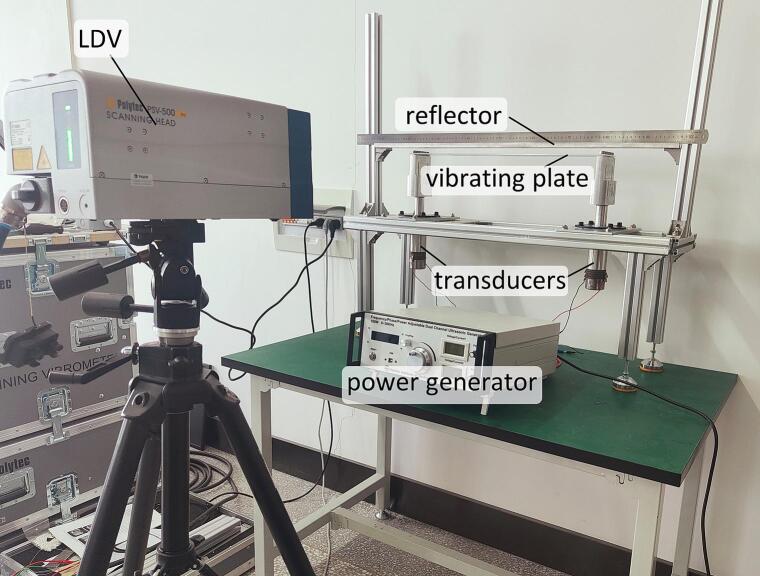
Photograph of the experimental equipment used for measuring acoustic field and for transporting small balls.

Fig. 5 illustrates the setup used for measuring the acoustic field and the dual-support transducer structure used for levitation and transportation. The dual-support transducer structure comprises two transducers fixed on the support structure, along with a vibrating plate connected to it. A flat reflector was positioned to the upper structure, opposite the vibrating plate, to form SWs in the *z* direction. A ruler was fixed to the reflector, and then used to reconstruct the acoustic field and record the position of the levitated object. The two transducers used have resonant frequencies of 19.905 kHz and 19.921 kHz, respectively. The vibrating plate was fabricated from aluminum (type 6061) and has the following material properties: elastic modulus E = 68.9 GPa, density *ρ* = 2750 kg/m^3^, and Poisson's ratio ν = 0.33. During the transportation process, the transducer was driven by a power generator (previously developed by the authors [29] – dual channel, 200 W, 0–120 V, 0–100 kHz, 0-360°).

To measure the sound pressure of the TW and 2D-SW acoustic field distributed between the vibrating plate and the reflector non-invasively, a Laser Doppler Vibrometer (LDV) was chosen [30]. When the laser beam from the LDV passes through the acoustic field, and where the refractive index, *n,* of the air was changed by the sound pressure, the optical path of the laser beam was modified as a result. This change in the optical path, *Δl*, can be considered as equivalent to the vibrational displacement, which could then be detected by using the LDV. As a result, the relationship between the velocity obtained from the LDV, *v*_LDV_, and the sound pressure, *P*, of the SW or TW acoustic field, can be stated to be as follows [30]:

(7) $$v_{\mathrm{LDV}}=\frac{2\pi f}{c_{0}^{2}\rho_{0}}\cdot \frac{n-1}{n}PS$$

where it can be noted that S represents the overlap of the laser and the acoustic field, while *c_0_* and *ρ_0_* are the velocity and density of the air, respectively. Therefore, according to Eq. (7), the amplitude of the sound pressure, *P*, can be seen to be directly proportional to the vibrational velocity, which can be measured by the LDV, *v*_LDV_.

In the following experiments, the vibrational displacement, *v*_LDV_, which is seen to be equivalent the change in optical path caused by the acoustic field was measured by using a scanning LDV (PSV-500-B type, Polytec). An extra single-point LDV (Model OFV-303, Polytec) was also connected to provide the required phase reference signal, because the phase of *v*_LDV_ is required to reconstruct the TW/2D-SW field.

## 5 Experimental verification of TW and 2D-SW transportation

As discussed in Section II, the dual-supported transducer structure used offers two distinct modes of transportation. In the TW mode, continuous movement of objects within the acoustic field was achieved by setting a specific value of θ suitable for high-speed transportation. On the other hand, the 2D-SW transportation mode allows for precise control over object movement. By adjusting the value of θ, the standing wave node can be accurately shifted, thus facilitating the step-by-step movement of the object and so this mode enables precise positioning and control of the position of the object. The effectiveness of both modes was verified experimentally, as discussed below.

### 5.1 Acoustic transportation experiments using TW

A. Measurement of the TW acoustic field

**Fig. 6:**
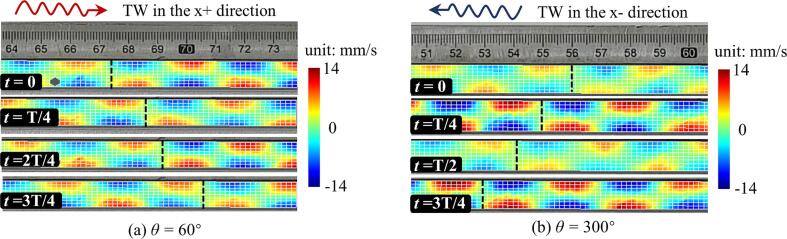
Vibrational velocity (measured using the LDV) of the TW acoustic field between the vibrating plate and the reflector (where f = 18.407 kHz, and Vpeak-peak = 80 V).

To measure the TW acoustic field between the vibrating plate and the reflector experimentally, a vibrating plate (with a length of 290 mm and a width of 60 mm) was selected, and the distance, *h*, between the vibrating plate and the reflector was set to 10.3 mm, this being approximately 0.5 times the wavelength in air. The peak-to-peak voltage and the frequency of the power generator were set to 80 V and 18.407 kHz, respectively. Using the configuration illustrated in Fig. 5, the results of the TW acoustic field varying with time and measured are as shown in Fig. 6. It can be noted that the TWs were observed when the value of θ was adjusted to be 60° and 300°, as illustrated by Fig. 6(a) and (b). In this way, the TW acoustic field used enables the rapid transportation of small objects.

B. Bidirectional TW transportation experiment

A TW transportation experiment was conducted, using the same configuration but except for an increase in the excitation voltage (to V_peak-peak_ = 120 V), this being chosen to increase the sound pressure of the acoustic field. Polystyrene spheres with a diameter of 3 mm were chosen and the spheres were released near the middle of the vibrating plate, with their motion trajectories recorded using a video camera (type ILCE-6400, Sony).

**Fig. 7:**
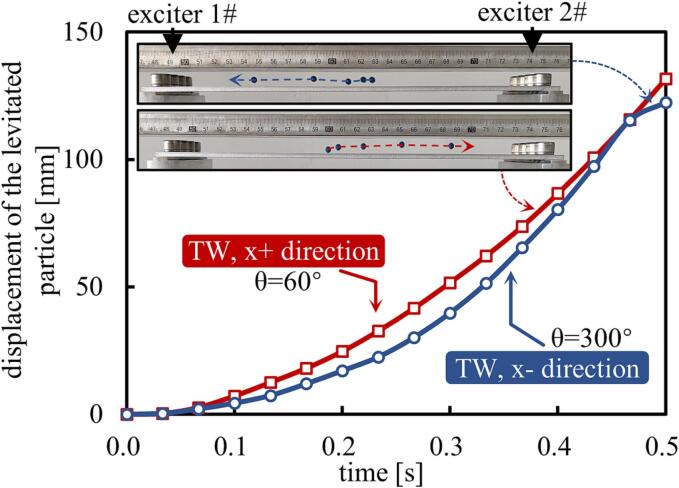
Relationship between the transport position of the polystyrene spheres and time in the TW mode (where *θ* = 60° and 300°, *f* = 18407 Hz, and V_peak-peak_ = 120 V). (This video is available as supplementary video 001 and video 002).

The trajectory of the position of the spheres was obtained from the experiment and this is shown in the upper right corner of Fig. 7. Here the images are superimposed at intervals of 0.1 s, over a period between 0 s and 0.5 s. The red and blue trajectories, representing θ values of 60° and 300°, respectively, show the opposite transportation directions and it is clear that the direction of the TW transportation is consistent with the direction of the TWs in the acoustic field.

Fig. 7 shows the displacement of the sphere relative to its initial release position, monitored as a function of time. It can be seen from the figure that the sphere experienced continuous acceleration throughout a time duration of 0.5 s, until it collided with the bolt positioned at the end of the vibrating plate.

The sphere traveled 131.7 mm (in the *x* + direction, θ = 60°) and 122.4 mm (in the *x* - direction, θ = 300°), taking times of 0.5 s and 0.47 s, respectively, where the maximum speeds achieved reached 480 mm/s and 540 mm/s. This result indicates that the TW transportation mode allows the fast movement of objects over long distances. However, it is important to note that some instability in the movement is induced in the TW transportation mode, and thus it is difficult accurately to control the position of the object.

### 5.2 Acoustic transportation experiments by programming θ

A. Measurement of the 2D-SW node position

Compared to the TW mode, the 2D-SW mode uses variations in the value of θ, instead of time variations, to control the position of the SW nodes, in that way to allow the sphere to remain levitated at a specific position. To investigate the relationship between the movement of the SW sound pressure node and the change of θ, measurements were made using the same device shown in Fig. 3. The vibrating plate used had a length of 580 mm and a width of 60 mm. The power generator was set to an 80 V peak-to-peak voltage and a frequency of 18.214 kHz and in order to avoid the formation of pure TWs, the temporal phase shifts were varied from 0° to 360° (in intervals of 60°).

**Fig. 8:**
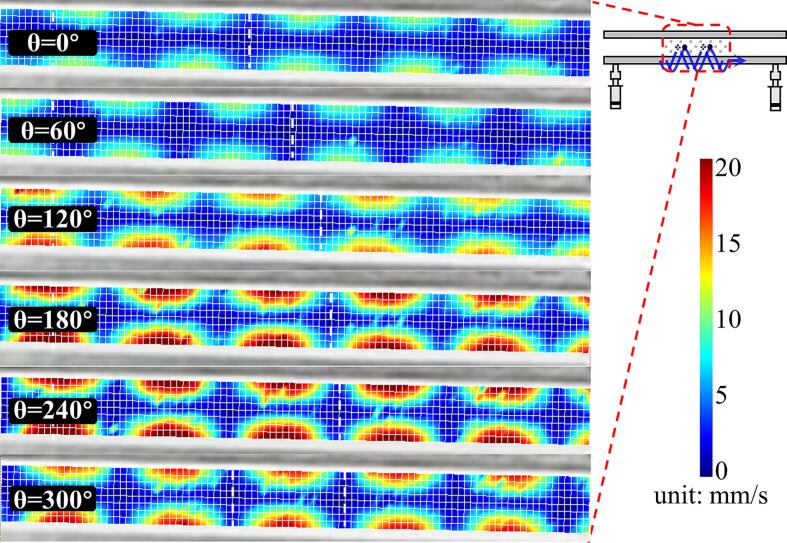
Distribution of the 2D-SW acoustic field obtained by using the LDV, illustrating the position of SW nodes changing with θ (where *f* = 18214 Hz, and *V*_peak-peak_ = 80 V).

The results of the measurement of the middle part of the acoustic field (where 240 mm < *x* < 340 mm) are as shown in Fig. 8. The scanning length (in the figure) was approximately 100 mm, as positioned near the center of the acoustic field. The white vertical dotted line shown represents the position of the SW node in the *x*-direction and when the value of θ changes from 0° to 300°, the node position gradually moves towards the *x* + direction, where the distance involved is approximately one wavelength (∼37 mm). The experimental results obtained here show that by adjusting θ, continuous transportation using the 2D-SW acoustic field could be realized.

B. Transportation experiment using 2D-SW

**Fig. 9:**
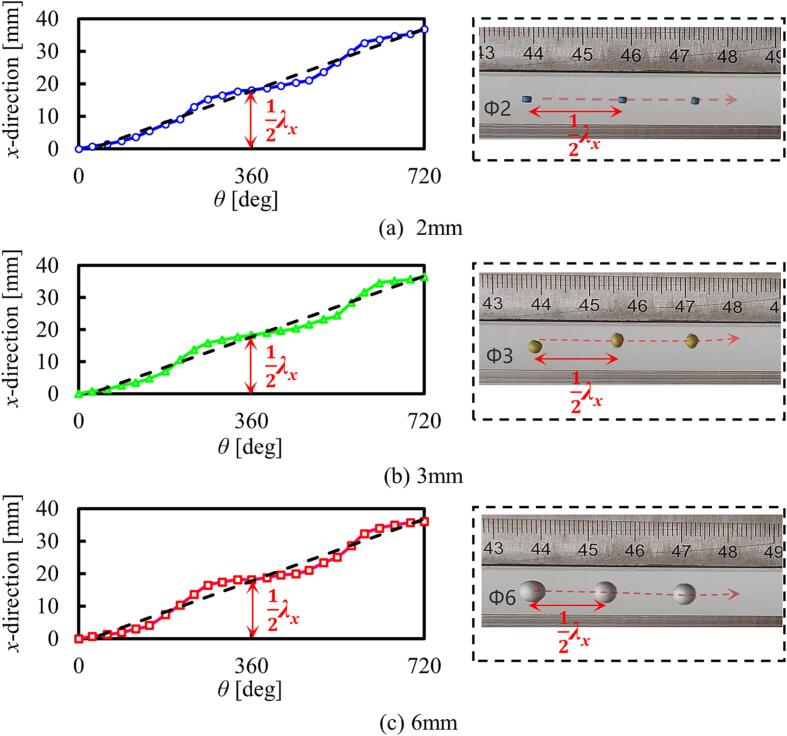
Relationship between the value of θ and the distance of transportation of the polystyrene spheres of different sizes (where *f* = 18214 Hz, and *V*_pp_ = 80 V).

In order to verify experimentally the effectiveness of the use of the 2D-SW mode, transportation experiments were conducted using polystyrene spheres with diameters of 2 mm, 3 mm, and 6 mm. By changing the temporal phase shift by 720° (i.e. twice 360°), a sphere with a 2 mm diameter was observed to move approximately 36.8 mm in the *x* + direction, this being close to the value of the wavelength of the acoustic field on the vibrating plate. Similarly, for the spheres with diameters of 3 mm and 6 mm, the distances moved by the spheres did not show any significant changes, under the same experimental conditions, and the relationship between these distances the spheres travelled and θ are shown in the left-hand column of Fig. 9. It is clear that the distances of the spheres move are approximately proportional to the value of θ, with values of 0.51 mm/°, 0.507 mm/°, and 0.501 mm/° for the spheres with diameters of 2 mm, 3 mm, and 6 mm, respectively. Additionally, the illustration in the right column of Fig. 9 shows a photograph of the positions of the levitation of these polystyrene spheres, with different diameters (where the images are superimposed at values of θ of 0°, 360°, and 720°). The results obtained demonstrate that the distances moved of the spheres was consistent with the value of θ used, further supporting the effectiveness of the 2D-SW mode for precise object positioning.

It can be noted that when θ has a value of ∼ 180° × (2n-1), the displacement motion of the sphere is slower, compared to using other phase shifts. This phenomenon was caused by the presence of the TW components which can be seen from Eq. (5). The existence of the TW components affects the position of the spheres, leading to a deviation from the position of the SW node (which has a linear relationship with θ). Consequently, as is shown in Fig. 9, as θ increases from 0° to the TW phase value, θ_1_, and then on to 180°, the offset of the position starts increasing, due to the augmentation of the TW component, and then decreases with the disappearance of the TW component.

Additionally, during the 2D-SW transportation process, oscillations and slight fluctuations near the equilibrium position may occur and affect the levitated object. This was caused by interference from external air effects and by nonlinear oscillations of the ultrasonic transducer and these are factors which potentially introduce errors into the measurement.

**Fig. 10:**
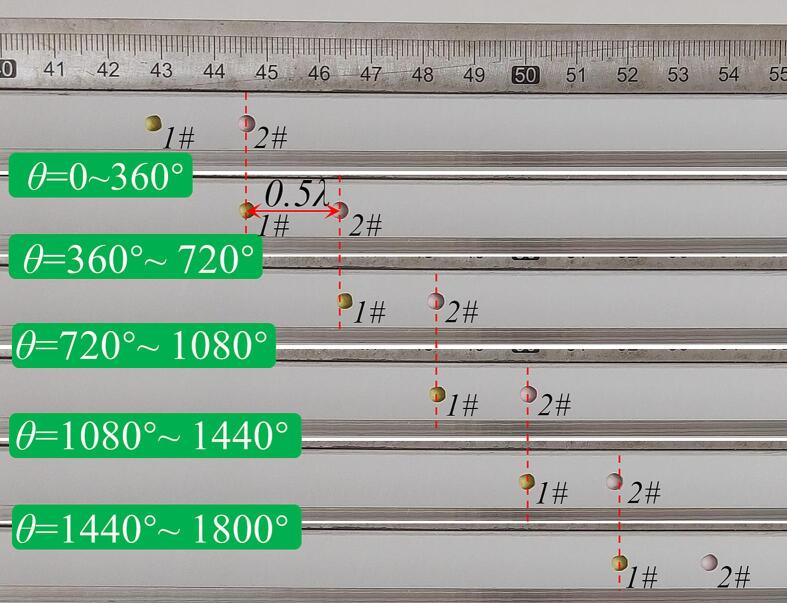
Photograph of experiments showing simultaneous transportation of two polystyrene spheres by adjusting the value of θ (where 3 mm diameter, *f* = 18.214 kHz, V_peak-peak_ = 80 V).

Moreover, the effectiveness of the 2D-SW transportation mode as a means of levitating and transporting multiple objects simultaneously, by adjusting the value of θ, has been verified in the experiments conducted. In this study, two polystyrene spheres, with a diameter of 3 mm, were initially placed at adjacent nodes in the SW acoustic field (with an initial value of θ = 0°). As θ was adjusted from 0° to 360°, the two spheres moved approximately 18 mm, a distance which corresponds to about 0.5 times the wavelength on the vibrating plate, determined in accordance with Eq. (7). To achieve a longer transportation distance, θ was further adjusted by 1800° (i.e. five times 360°), resulting in a transportation length of 90 mm, which is approximately 2.5 times the wavelength on the vibrating plate. This process was accomplished within 43 s by programming the power generator to change θ automatically, as shown in Fig. 10, (and the transportation of the three polystyrene spheres is shown in Video 003 in the Supplementary data). Thus, the successful transportation of objects over a longer distance using the 2D-SW mode has been demonstrated in the experiment carried out and reported.

## 6 Conclusion

In this work, it has been shown that the 2D-SW and TW transportation modes were both successfully employed in the dual-transducer support structure illustrated. To understand further the principles of acoustic transportation by adjusting θ used in this work, θ-related expressions for the TW component and the positions of the SW nodes were deduced and as a result, both the TW and the 2D-SW modes have been described well theoretically and simulated through using finite element analysis. To verify the effectiveness of the use of the two modes, non-contact measurements of the TW and SW acoustic pressure distributions were performed and the results reveal that no matter which mode was used, SWs in the *z* direction were formed and could be employed to levitate the objects between the vibrating plate and the reflector. The 2D-SW field was generated with θ-adjustable node positions, while TW fields were formed at a specific pair of values of θ.

In the experiments carried out, it was demonstrated that polystyrene spheres (with diameters ranging from 2 mm to 6 mm) were transported successfully. The speed achieved reached 2 mm/s using the 2D-SW mode, where precise control over the position of the spheres was achieved, despite the low transportation speed. In contrast, in the TW mode, the spheres were transported at a much higher speed (of 540 mm/s), making this approach suitable for high-speed transportation scenarios, such as are seen in the large-scale production of pharmaceutical products. As a result, the experiment results obtained have revealed several advantages in the dual-support transducer structure over simple transducer arrays, such as allowing for more precise control of the levitated position of the objects using the 2D-SW mode, as well as enabling long-distance and high-speed transportation at a lower cost, using the TW mode.

## Funding

This work was supported by the National Natural Science Foundation of China [grant numbers 52275014]. Grattan and Sun acknowledge support from the Royal Academy of Engineering and the Royal Society in the UK.

## CRediT authorship contribution statement

**Guanyu Mu:** Methodology, Writing – original draft. **Huijuan Dong:** Conceptualization, Investigation, Writing – review & editing. **Tong Sun:** Visualization, Investigation. **Kenneth T.V. Grattan:** Investigation, Writing – review & editing. **Zhiguang Wu:** Writing – review & editing. **Jie Zhao:** Supervision, Resources.

## Declaration of competing interest

The authors declare the following financial interests/personal relationships which may be considered as potential competing interests: Huijuan Dong reports financial support was provided by National Natural Science Foundation of China.

## Data Availability

Data will be made available on request.
